# Fast pH‐Driven Solubilization Method of Realgar (As_4_S_4_) to Reduce the Toxicity of Arsenic [As(III)] for Medicinal Purposes

**DOI:** 10.1002/advs.202502740

**Published:** 2025-04-24

**Authors:** Bojana Lucic, Douglas Santana Franciscato, Helton Pereira Nogueira, Lara Gallucci, Alceu Totti Silveira Junior, Asmaa Mohamed Ismail, Millie Robinson, Teresa Dallinger, Claudia Gutfleisch, Jochen Kurz, Maytê Toledo, Jessica Dias da Silva Ferraz, Mohammad Tarek, Danilo Dias, Ricardo Sobhie Diaz, Mahmoud ElHefnawi, Mattia Forcato, Hugo P Monteiro, Marina Lusic, Iart Luca Shytaj, Andrea Savarino

**Affiliations:** ^1^ Department of Infectious Diseases Integrative Virology Heidelberg University 69120 Heidelberg Germany; ^2^ German Center for Infection Research 69120 Heidelberg Germany; ^3^ Institute of Chemistry University of São Paulo São Paulo 05508‐220 Brazil; ^4^ School of Cellular and Molecular Medicine University of Bristol Bristol BS8 1TD UK; ^5^ Spectroscopy Department National Research Centre 33 El Bohouth Street Dokki Giza 12622 Egypt; ^6^ Center for Infectious Diseases Medical Microbiology und Hygiene University Hospital Heidelberg 69120 Heidelberg Germany; ^7^ Department of Biochemistry Center for Cellular and Molecular Therapy Federal University of São Paulo São Paulo 04021‐001 Brazil; ^8^ Infectious Diseases Department Federal University of São Paulo São Paulo 04021‐001 Brazil; ^9^ Clinical Hematology Department Armed Forces College of Medicine (AFCM) Cairo Governatorate Heliopolis 11774 Egypt; ^10^ Informatics and Systems Department National Research Centre 33 El Bohouth Street, Dokki Giza 12622 Egypt; ^11^ Department of Molecular Medicine University of Padova Padova 35122 Italy; ^12^ Department of Infectious Diseases Italian Institute of Health Rome 00161 Italy

**Keywords:** Acute Promyelocytic Leukemia, Arsenic Trioxide, HIV, PML, Realgar

## Abstract

Acute promyelocytic leukemia (APL) accounts for 5–15% of acute myeloid leukemia cases. It is typically characterized by the (15;17) chromosomal translocation, producing the pathogenic retinoic acid receptor (RAR) alpha/promyelocytic leukemia (PML) fusion protein. Recently, remission of APL has been achieved using the first chemotherapy‐independent oral drug regimen in anticancer therapy, consisting of all‐trans retinoic acid (targeting RARalpha) and the arsenic sulfide realgar (targeting PML). However, clinical adoption of realgar and the characterization of its active breakdown products have been hampered by its poor solubility. Here, a scalable pH/temperature‐based process is described that partially mimics gut transition, achieving fast and reproducible solubilization of realgar. Six different spectroscopic and spectrometric techniques are employed to investigate solubilized realgar. Furthermore, it is shown that solubilized realgar targets PML, displaying wider in vitro therapeutic indices and lower off‐target effects than arsenic trioxide, the current APL standard of care. Moreover, in line with evidence of an interplay between PML and HIV persistence, solubilized realgar can disrupt HIV latency, the main barrier to an HIV/AIDS cure, in CD4 T cells of people living with HIV. These findings may open avenues for streamlining realgar solubilization and designing less toxic, orally administrable arsenic‐based therapies.

## Graphical abstract

Arsenic trioxide transformed acute promyelocytic leukemia therapy and shows promise against HIV. A new method to solubilize the mineral realgar produces different arsenic species depleting the main arsenic target, nuclear PML protein, and retaining effects on leukemic and HIV reservoir cells, while minimizing off‐target damage. These results highlight solubilized realgar as a potentially safer, orally available alternative for arsenic‐based treatments.

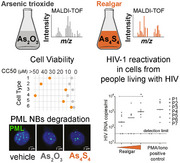

## 1 Introduction

There is extensive research ongoing on arsenic as a therapeutic agent, also prompted by recent results broadening the possible spectrum of activity for arsenic trioxide (As_2_O_3_).^[^ ^1^, ^2^ ^]^ Apart from its main indication against acute promyelocytic leukemia (APL), arsenic trioxide has become an investigational agent against several neoplastic and non‐neoplastic diseases, including human immunodeficiency virus (HIV)/AIDS,^[^ ^1^, ^2^ ^]^ for which it is currently being investigated in a clinical trial (https://clinicaltrials.gov/study/NCT03980665). Specifically, arsenic trioxide has been suggested to disrupt the persistence of HIV as latent, integrated DNA in the host cells,^[^ ^1^, ^2^, ^3^, ^4^ ^]^ which is regarded as the main obstacle to overcome in achieving a cure for the infection.^[^ ^5^ ^]^ The toxicity of arsenic trioxide, however, results in a narrow therapeutic index, which limits many of its potential applications.^[^ ^6^ ^]^

Tetrarsenic tetrasulfide (As_4_S_4_), also known as realgar, was recently approved for treatment of APL as a potentially safer, oral alternative to arsenic trioxide^[^ ^7^ ^]^ Realgar has been used for centuries in ancient China as a remedy for various ailments, including psoriasis, burns, and abdominal pain.^[^ ^8^ ^]^ The first mention of the medical use of realgar appears in the Emperor's Materia Medica, dating back to the 2^nd^ century BCE.^[^ ^6^ ^]^ Its current therapeutic application stems from the results of a phase III clinical trial that compared the combination of oral all‐trans retinoic acid (ATRA), which is active against APL, with either a preparation containing realgar or standard‐of‐care intravenous arsenic trioxide. The trial demonstrated the non‐inferiority of the realgar‐containing preparation.^[^ ^7^ ^]^

Unlike other oral arsenic derivatives such as orpiment (As_2_S_3_), realgar in its crystalline form does not undergo covalent polymerization but nevertheless is characterized by very poor water solubility, which significantly limits its use as a medication.^[^ ^9^ ^]^ For this reason, only 0.1–4% of the total realgar that passes through the digestive system is solubilized and absorbed.^[^ ^10^, ^11^ ^]^ Consequently, realgar is administered in a high‐dosage preparation of 60 mg kg^−1^, containing ≈400 times the arsenic equivalent of the typical intravenous arsenic trioxide preparation (0.15 mg kg^−1^).^[^ ^7^ ^]^ The poor solubility of realgar has hindered research into its full therapeutic potential as an alternative to arsenic trioxide and into the characterization of its solutes. One study concluded that the ultimate fate of realgar in vivo is its transformation into dimethylarsinic acid, a product of arsenic detoxification.^[^ ^12^ ^]^ In addition, Zhang et al. pioneered the characterization of realgar ingested for non‐medical purposes (i.e., realgar wine) by using a simulated gastrointestinal system to investigate the leaching of arsenic in alcohol.^[^ ^13^ ^]^ However, only the oxidation state of the released arsenic, and not the molecular structures containing the arsenic ions, was characterized.^[^ ^13^ ^]^ Overall, the solutes derived from realgar that are absorbed in the gut and responsible for its therapeutic effects remain largely unidentified at present.^[^ ^14^ ^]^

An optimal method for leaching realgar should result in active substances similar to those released in the gut after mineral ingestion, likely improving bioavailability. Additionally, it should be scalable, cost‐effective, fast, and reproducible. Developing one such method could expand the therapeutic potential of realgar by allowing for a more controlled administration of the drug, limiting the risk of toxicity, and optimizing the use of the extracted mineral, with beneficial industrial and environmental repercussions. In particular, limiting the amount of arsenic excreted would reduce the chronic exposure of aquatic life to low‐level arsenic, which may result in genotoxicity.^[^ ^15^ ^]^

One approach to improving the bioavailability of realgar is through its incorporation into water‐soluble nanoparticles, which has shown promising results in mice.^[^ ^16^ ^]^ On the other hand, effective solubilization of realgar could also be achieved through a bioleaching process. Specifically, this protocol uses chemical reactions catalyzed by *Acidithiobacillus ferrooxidans* and/or *A. thiooxidans* over a period of 20 days or more.^[^ ^17^, ^18^ ^]^ This method achieved complete solubilization of arsenic from realgar, but its complexity and slow pace partially limit its industrial applicability. Moreover, reactions catalyzed by exogenous prokaryotic organisms may yield solutes that differ significantly from those produced through the spontaneous leaching of arsenic in the human digestive system. For instance, one of the products of the *Acidithiobacillus*‐catalyzed reactions is arsenic in the form of arsenate, As(V), which is not as biologically active as its trivalent counterpart. In particular, As(V) does not induce degradation of the promyelocytic leukemia protein (PML), a key target for the anticancer and anti‐HIV latency effects of arsenic.^[^ ^1^, ^7^, ^9^ ^]^

A publication by Berzelius, dating back to 1826, suggested an alternative and more rapid method for realgar solubilization.^[^ ^19^ ^]^ This method involves boiling realgar‐containing water in a highly alkaline environment, a simple process that might also partially mimic some of the reactions occurring in the alkaline tracts of the human intestine.^[^ ^20^ ^]^ However, the lack of modern analytical techniques at that time limited the characterization of this method, and the biological effects of the solution have never been tested.

Here, we present a modified version of Berzelius's method. Our conditions achieve solubilization of realgar by sequential exposure to acidic and alkaline solvents, followed by a final heating step. Our pH conditions were selected to increase solubilization of realgar, but they also partially mimic the passage through the stomach (acidic) and parts of the intestine (slightly alkaline)^[^ ^21^ ^]^ potentially allowing to infer the breakdown of solid realgar after ingestion. The solubilization protocol adopted here is relatively fast (72 h), while requiring only inexpensive reagents and mild temperature conditions. Noteworthy, the solution retains biological activity, targeting PML/APL and promoting HIV latency disruption in vitro, potentially opening new avenues in the therapeutic use of oral derivatives of arsenic.

## 2 Results

### 2.1 Morphological Changes During Realgar Solubilization in Acidic and Alkaline Solutions

**Figure 1:**
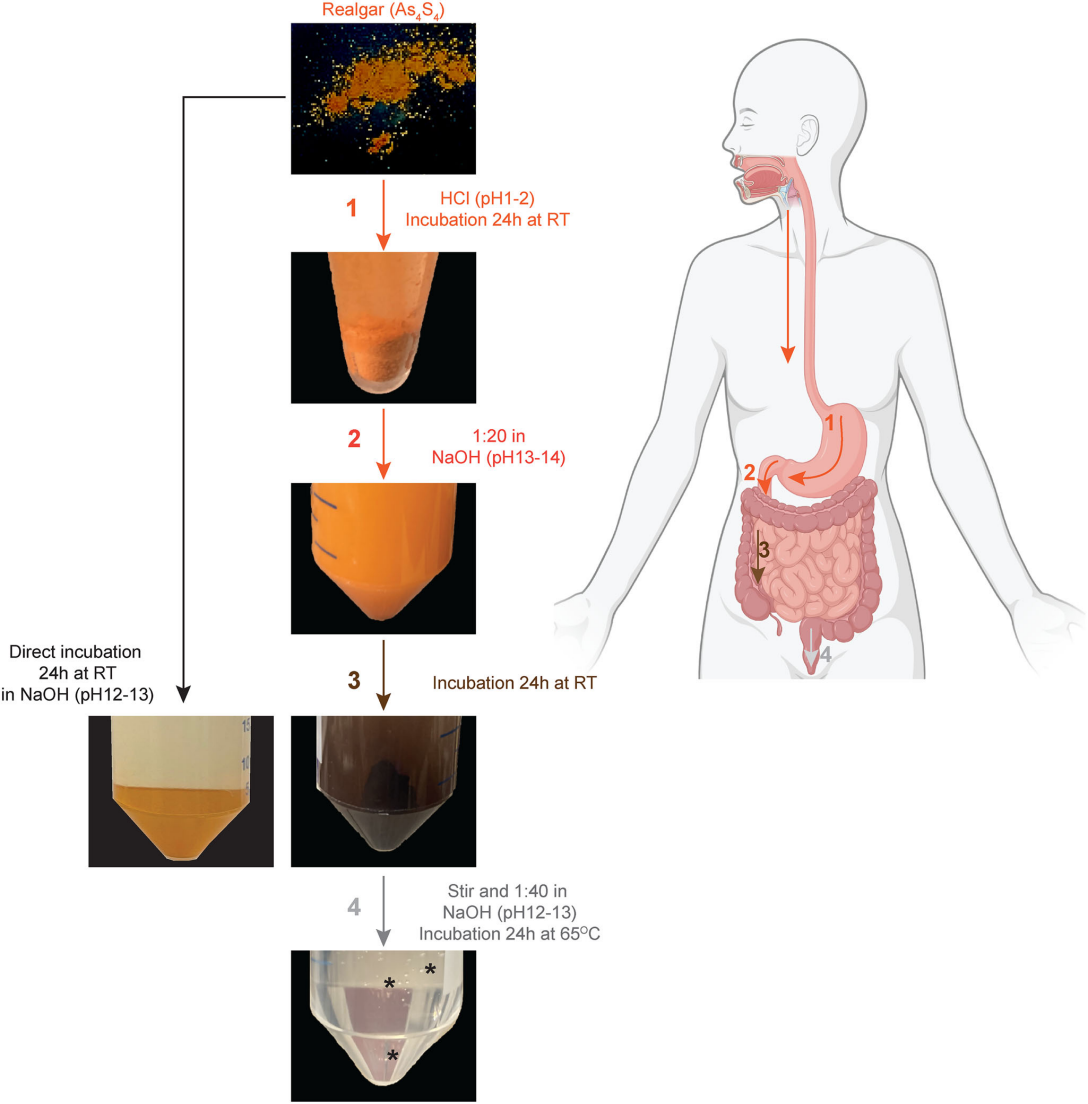
Realgar solubilization protocol. Step 1. Incubation of the realgar powder in HCl (pH 1–2); step 2. 1:20 dilution of the product of step 1. in an NaOH solution (pH 13–14) and effects of its incubation (step 3) for 24 h; step 4. 1:40 dilution of the product of step 3. in an NaOH solution (pH 12–13) and incubation for 24 h at 65°. The left panel in step 3 shows the effect of direct incubation of realgar powder in an NaOH solution (pH 12–13). Asterisks indicate the formation of bubbles in step 4. A small amount of black precipitate could be detected at step 4 and could be removed by centrifugation. The backgrounds of the pictures taken for each solubilization step were replaced with a uniform black box in Adobe Illustrator to facilitate comparisons between conditions. The right panel illustrates a schematic representation of the digestive system (created with BioRender.com), showing, in parallel to the organs, the pH conditions partially mimicked during the different solubilization steps. Note that the alkalinization occurring in the jejunum and the lower intestine has been exaggerated in our experimental set up to obtain near complete solubilization, which does not occur in the organism.

A step‐by‐step description of the procedure that we followed to solubilize realgar is depicted in **Figure** **1** and detailed in the Methods section. The initial incubation in an acidic HCl solution (pH 1–2) led to a poorly solubilized, orange‐colored suspension (Figure 1, step 1). The dilution of this suspension in a basic (pH 13–14) NaOH solution resulted in dark‐brown material (Figure 1, steps 2–3). The size of the crystals at this time point was > 100 µm, as determined by filtration. Following further dilution in a basic solution at a lower pH (pH 12–13) and overnight incubation at 65 °C, a transparent, colorless solution was obtained after thorough mixing, during which bubbling was observable (Figure 1, step 4). A small amount of black particulate matter was present and could be removed by centrifugation. The overall solubilization process could be reproducibly applied using a 100 mg input of realgar, resulting in a 0.125 mg mL^−1^ solution in the final step. The most pronounced transformation of realgar was observed after incubation with NaOH at pH 13–14 (steps 2–3). This was further highlighted by the fact that direct incubation of realgar powder with NaOH at a lower pH (12–13) failed to induce a color change (Figure 1, left arrow).

### 2.2 FT‐IR, ED‐XRF, and Raman Spectroscopy Analyses of Solubilized Realgar

We initially evaluated the chemical changes of realgar during the different solubilization steps using three different approaches. A preliminary estimate of arsenic solubilization was obtained by FT‐IR, with the analysis being focused within a spectrum in the range of 4000–400 cm^−1^ (Figure S1, Supporting Information). The analysis showed a gradual change in the spectra following each of the realgar transformation steps. The transformation of realgar was accompanied by the appearance of a smaller peak at 444 cm^−1^, starting from the first incubation (Figure S1, Supporting Information). Moreover, specific As‐O related vibrations might be inferred from the peak at 887 cm^−1^ (SpectraBase Spectrum ID: 2me3I8pKhLl).

We further investigated the products of realgar solubilization using ED‐XRF and Raman spectroscopy. The semi‐quantitative results of the ED‐XRF analysis showed that arsenic leached from realgar starting from the first reaction in HCl (Figure S2, Supporting Information). This finding is in line with the ED‐XRF spectra of the solubilized material derived from each reaction, indicating the presence of arsenic atoms in solution (Figure S2, Supporting Information).

To better characterize the chemical nature of solubilized realgar, we used Raman spectroscopy (Figure S3, Supporting Information). The results of this analysis showed that the final solubilization product of realgar (step 4 in Figure 1) was different from the reference Raman spectra of both sodium arsenate (SpectraBase Compound ID: DcgQIspdvbW) and sodium arsenite (SpectraBase Compound ID:606PKSOrnxm) (Figure S3A,C,D, Supporting Information),^[^ ^22^ ^]^ showing peaks in the 400s cm^−1^, which are not present in the reference spectra of the As‐O derivatives. A major peak is observed at 458 cm⁻¹ (Figure S3A, Supporting Information), previously calculated to correspond to S‐S stretches within an arsenic sulfide cluster (As₂S₅).^[^ ^23^ ^]^ However, peaks in the 400s cm⁻¹ can also be attributed to As‐S stretches when different moieties are bound to the arsenic atom.^[^ ^24^ ^]^ In this case, the band might be related to the aforementioned one appearing in FTIR at a similar frequency (Figure S1, Supporting Information). Moreover, as described for spectra obtained with equimolar S/As ratios in alkaline conditions, our Raman spectra displayed the main bands at the 500s and 800s cm^−1^, previously attributed to thioarsenite.^[^ ^25^ ^]^ However, the exact interpretation of the Raman spectra of thioarsenites has remained a matter of debate through the decades.^[^ ^25^, ^26^, ^27^ ^]^

Generation of heterogeneous chemical species was further supported by the analysis of the dark particulate matter appearing in low amounts at the end of the solubilization process and displaying peaks in a wide range of wavelengths (up to 1200 cm^−1^) (Figure S3B, Supporting Information). The main peaks/shoulders in the low wavenumber range (100–400 cm^−1^) partly resembled the spectra previously described for metal arsenic.^[^ ^28^ ^]^ The preparation showed a main peak at 198 cm^−1^, a minor peak at 228 cm^−1^ and a shoulder at 238 cm^−1^. These are also reminiscent of peaks found in pararealgar but not present in natural realgar or orpiment.^[^ ^29^ ^]^ Although the attribution of these features has so far been unclear, they may be due to metal‐metal‐metal angles, not present in realgar and orpiment. Some peaks at higher wavelengths could also be attributed to/influenced by As‐O vibrations (Figure S3, Supporting Information)^[^ ^30^ ^]^ or As‐Cl, similar to those observed with arsenite and chloroarsenite compounds.^[^ ^31^ ^]^ The presence of chlorine could explain some of the spectral features that would not be consistent with pure arsenic or sodium arsenide.^[^ ^31^ ^]^ Finally, the high wavenumber peak (between 1100 and 1200 cm^−1^) did support the presence of multiple components.

To sum up, the products of realgar transformation presented complex spectra consistent with a mixture of molecules likely including not only thioarsenites but also more complex structures.

### 2.3 Mass Spectrometry Characterization of Solubilized Realgar

**Figure 2:**
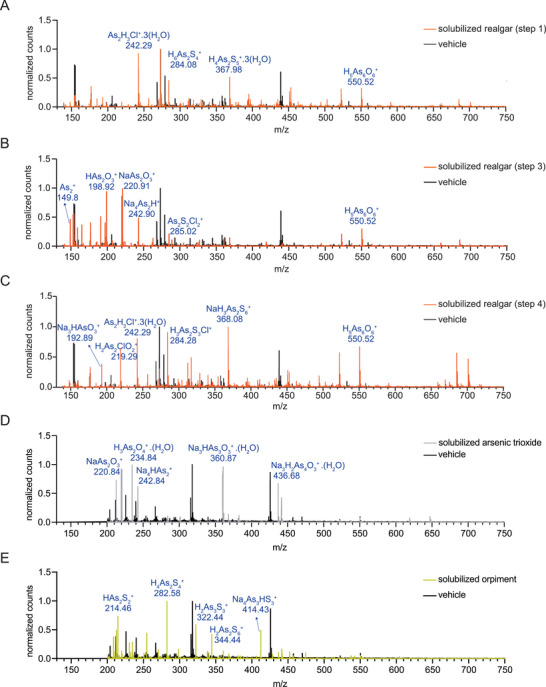
MALDI‐TOF spectra of the products of realgar solubilization. The products of each step of realgar solubilization (depicted in Figure 1) were analyzed by MALDI‐TOF. A) Spectrum obtained after the first incubation step in HCl. B) Spectrum obtained after the subsequent incubation in NaOH (pH 13–14). C) Spectrum obtained after the final incubation step in NaOH (pH 12–13). D,E) Spectra obtained after solubilization of arsenic trioxide (D) and orpiment (E) in NaOH (pH 12–13). The differences in the DHB peaks in (A,B,C) and (D,E) are due to the different diluents [aqueous solution of TFA (1%) and equivolume water acetonitrile 1:1/TFA (1%)] used in different runs and resulting in different crystallization of mass matrix. The tentative interpretation of the major peaks is shown above the corresponding peak. Panels D and E report the peaks above 200 m z^−1^ because of interference with the DHB signal. A basis for the molecular formulas that we propose can be found in refs.[25, 60, 61, 62, 63, 64, 65, 66, 67]

To investigate the solution further, we then proceeded to analyze the products of the different steps of realgar solubilization by two different mass spectrometry approaches. We first conducted an Electrospray Ionization (ESI) analysis, which, especially in the final solubilization product (step 4 of Figure 1), resulted in high m/z fragments that could not be immediately analyzed due to the large number of possible atomic interactions. We thus conducted a MALDI analysis coupled to time‐of‐flight (MALDI‐TOF) which produced lower m/z fragments. The MALDI‐TOF spectra showed an increasing molecular diversity following each of the steps of realgar solubilization (**Figure** **2**A–C). The heterogeneous composition of the final solution, as derived from this analysis, was in line with the results of the Raman analysis (Figure S3A, Supporting Information). In general, the process of realgar solubilization led to greater molecular diversity than that observed with arsenic trioxide and orpiment solubilized in NaOH. The final realgar solution (Figure 2C) displayed some peaks similar to those displayed by solubilized arsenic trioxide and orpiment, but also characteristic peaks with higher m/z (Figure 2D,E).

Overall, the mass spectrometry analysis, in line with the analyses reported in the previous paragraph, revealed a complex mixture of compounds. The biological activity of this solution thus warranted further investigation.

### 2.4 Comparison of the In Vitro Toxicity of Arsenic‐Containing Compounds

Arsenic‐containing compounds have been previously associated with off‐target cytotoxicity, potentially leading to damage in several organs, including the heart, skin, vascular endothelium, and gastrointestinal tract.^[^ ^32^ ^]^

We evaluated the cytotoxic effects of solubilized realgar in a panel of cell cultures of different origins, mimicking cell types potentially affected by arsenic toxicity, including the melanoma cell line A‐375, the human colorectal adenocarcinoma cell line SW480, primary endothelial cells (HUVEC) and primary cancer‐associated fibroblasts (HF002J cells). To assess the relative cytotoxicity of our realgar solution, we compared its effects with those of arsenic trioxide and orpiment, both solubilized in an NaOH solution at the same pH used in the final step of the realgar solubilization protocol (Figure 1). To facilitate the comparison among these three arsenic‐containing compounds and with the extant literature, we followed the convention of calculating molarity by considering the molecular formula of the input arsenic compounds (i.e., As_2_O_3_, As_4_S_4_, As_2_S_3_). Moreover, in parallel, we reported the amount of arsenic present in each of the three solutions, as calculated by ICP‐MS (Table S1, Supporting Information).

**Figure 3:**
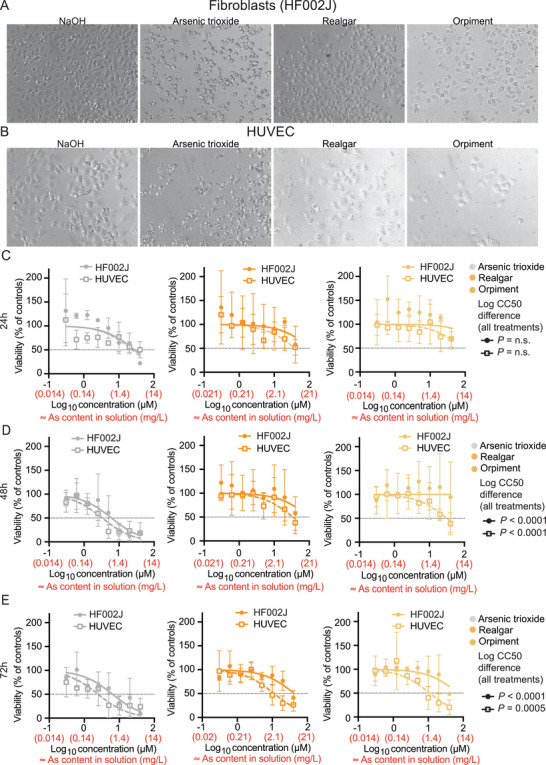
Adhesion and viability of primary fibroblasts and endothelial cells treated with arsenic‐containing compounds. Primary cancer‐associated fibroblast HF002J cells and human vascular endothelial cells (HUVEC) were treated with different concentrations of solubilized realgar, arsenic trioxide, or orpiment. Cells treated with NaOH (solvent) were used as controls. Cell adhesion (A,B) was monitored 24 h post‐treatment, as shown by representative microphotographs (200x) of cells treated with 20 µm of each arsenic‐containing compound. Cell viability (C–E) was measured by MTT assay 24 h (C), 48 h (D) or 72 (E) h post‐treatment. For each treatment condition, the relative viability was calculated by normalizing the absorbance values of cells treated with each arsenic‐containing compound to the absorbance of cells treated with NaOH. Half‐maximal cytotoxic concentration (CC50) values were calculated by non‐linear regression. Extra sum‐of‐squares *F* test was performed to assess differences in LogCC50 values between the inhibition curves. Data are depicted as mean ± SD. N = 3.

The results of MTT assay experiments showed that solubilized realgar was less cytotoxic than arsenic trioxide in the A‐375 and cell lines (Figure S4A,B, Supporting Information). These results were mirrored by primary cells where realgar had a reduced impact on the adhesion of HF002J fibroblasts and HUVECs after 24 h of incubation (**Figure** **3**A,B). Moreover, the half‐maximal cytotoxic concentration (CC50), i.e., the drug concentration at which each dose‐effect curve intersected 50% cell viability, was consistently higher for realgar compared to arsenic trioxide (Figure 3C–E) indicating a lower cytotoxicity of realgar. Solubilized orpiment displayed cytotoxicity levels comparable to those of realgar (Figure 3; Figure S4A,B, Supporting Information). Overall, a statistically significant divergence in CC50 values obtained when treating with different arsenic‐containing compounds was observed in cell lines and primary cells after 48 and 72 h of treatment (Figure 3D,E; Figure S4A,B, Supporting Information).

These data indicate that realgar is better tolerated by cell models mimicking targets of in‐vivo arsenic toxicity, as compared to arsenic trioxide, which is currently the most widely used arsenic‐containing drug.

### 2.5 In Vitro Targeting of PML Protein and APL Cells by Solubilized Realgar

The main molecular target of the therapeutic activity of arsenic trioxide is the PML protein and its related nuclear bodies (NBs). In line with this, arsenic trioxide has been shown to induce degradation of the PML/RARalpha fusion product in APL cells.^[^ ^33^ ^]^

**Figure 4:**
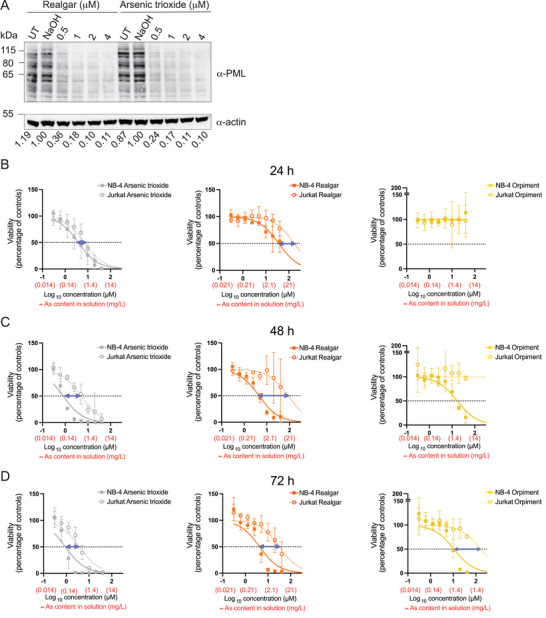
Targeting of PML and selectivity for APL cells by arsenic‐containing compounds. A) Western blot analysis of PML protein expression 72 h post‐treatment of NB‐4 cells. The numbers below each lane indicate the relative expression of PML, normalized to the housekeeping protein (actin) expression. B–D) The APL (NB‐4) and non‐APL (Jurkat E6.1) cell lines were left untreated or treated with serial dilutions of arsenic‐containing compounds or their solvent (NaOH). The relative viability of cells 24 h (B), 48 h (C), and 72 h (D) post‐treatment was assessed by MTT assay. Relative viability was calculated by normalizing the absorbance values of cells treated with each arsenic‐containing compound to the absorbance of cells treated with NaOH. Half‐maximal cytotoxic concentration (CC50) values were calculated by non‐linear regression. The blue arrows indicate the difference between CC50 values in NB‐4 versus Jurkat E6.1 cells. Data in B‐D are depicted as mean ± SD. N = 3.

To evaluate the in vitro therapeutic potential of our solubilized realgar product, we tested its ability to induce degradation of PML in NB‐4 cells, an APL cell line expressing PML/RARalpha.^[^ ^34^ ^]^ The results showed that solubilized realgar was able to deplete PML at submicromolar concentrations at 72 h post‐treatment (**Figure** **4**A). This effect was comparable to that of arsenic trioxide (Figure 4A).

Arsenic trioxide was previously associated with more potent targeting of NB‐4 cells, compared to other, non‐APL, leukemic cell lines, including Jurkat T cells.^[^ ^35^, ^36^ ^]^ We therefore used a similar experimental setup to evaluate the potential selectivity of solubilized realgar for NB‐4 cells compared to Jurkat E6.1 cells. Both cell lines were treated in parallel with solubilized realgar, arsenic trioxide, and orpiment for 24–72 h, and the relative viability (normalized to solvent, i.e., NaOH control) was estimated by MTT assay. Relative viability data were used to calculate a therapeutic index as the ratio between the CC50 values of Jurkat E6.1 cells and NB‐4 cells. As expected, all three arsenic‐containing compounds were more cytotoxic against NB‐4 cells (Figure 4B–D); however, our results showed that solubilized realgar displayed the best therapeutic index (Figure 4B–D; Figure S4C,D, Supporting Information). Specifically, realgar displayed more potent effects against NB‐4 cells compared to orpiment but was less toxic than arsenic trioxide against Jurkat E6.1 cells (Figure 4B–D). A general trend of higher selectivity against NB‐4 cells could be detected for solubilized realgar, when the analysis was extended to the other cell types tested with arsenic‐containing compounds (HF002J, HUVEC, SW480, A‐375) (Figure S4C,D, Supporting Information).

### 2.6 Distinct Transcriptomic Effects of Solubilized Realgar in APL and Non‐APL Cells

Beyond its effects on cytotoxicity, treatment with arsenic trioxide has been associated with broad effects on gene expression in NB‐4 cells, which have in turn been linked to its therapeutic efficacy.^[^ ^37^ ^]^ On the other hand, broad transcriptomic dysregulation in non‐APL cells could account for off‐target toxic effects of arsenic‐containing treatments.

To compare the transcriptomic modulation induced by arsenic‐containing compounds, we subjected NB‐4 cells and Jurkat E6.1 cells to RNA‐Seq analysis after treatment with solubilized realgar, arsenic trioxide or orpiment. To exclude potential biases due to the generally higher cytotoxicity of arsenic trioxide (Figure 4B–D), we used a short‐term (48 h) treatment at a well‐tolerated, submicromolar (0.5µm) concentration in both cell lines that is, however, sufficient to induce PML depletion (Figure 4A). Cells incubated with solvent (NaOH) were used as controls.

**Figure 5:**
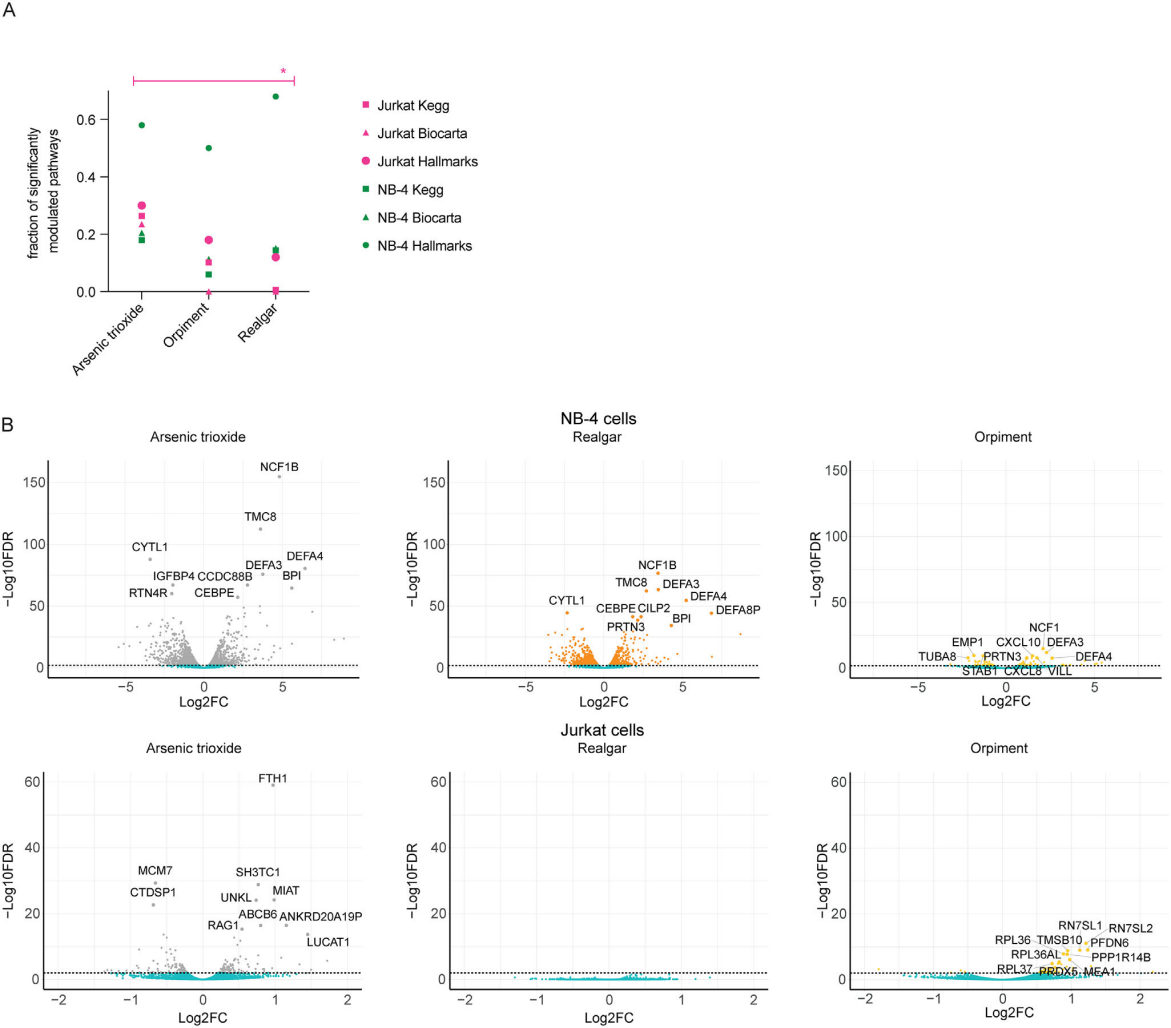
Transcriptomic effects of arsenic‐containing compounds in APL and non‐APL cells. Jurkat E6.1 (non‐APL) and NB‐4 (APL) cells were treated for 48 h with 0.5 µm of either realgar, arsenic trioxide, orpiment, or vehicle (NaOH) and subjected to RNA‐Seq. A) Fraction of pathways significantly modulated by each drug, as compared to the solvent NaOH control in Jurkat E6.1 cells (purple) and NB‐4 cells (green). Significantly modulated pathways were identified by GSEA (FDR ≤ 0.25 as in^[^ ^38^ ^]^), and their fraction was calculated based on the total number of pathways in each gene set repository. Differences in the fractions of pathways significantly modulated by each drug were calculated by one‐way ANOVA followed by Tukey's post‐test. The test was preceded by an appropriate transformation to restore normality and account for the null number of modulated pathways in the realgar and orpiment Biocarta dataset: *Y’ = Log10(y + 1)*. B) Volcano plots of genes modulated by treatment with arsenic‐containing compounds, as compared to vehicle (NaOH) control. Gene fold change and FDR levels were calculated using the edgeR package. Grey, orange, and yellow dots indicate genes with FDR < 0.01 (i.e., ‐Log10 FDR > 2) in arsenic trioxide, realgar, and orpiment treatment conditions, respectively. The names of the top ten genes with FDR < 0.01 are depicted.

We first evaluated alterations in pathway expression by performing Gene Set Enrichment Analysis (GSEA) using the annotated gene set repositories Kegg, Biocarta, and Hallmark, and setting FDR ≤ 0.25 as the significance threshold, as previously described.^[^ ^38^ ^]^ For all gene sets, the analysis showed that treatment of NB‐4 cells with arsenic‐containing compounds significantly altered the expression of comparable numbers of pathways (Biocarta = 20, Kegg = 24, Hallmark = 34 for realgar; Biocarta = 27, Kegg = 30, Hallmark = 29 for arsenic trioxide; Biocarta = 15, Kegg = 10, Hallmark = 25 for orpiment) (**Figure** **5**A; Table S2, Supporting Information). Conversely, the number of pathways significantly modulated in Jurkat E6.1 cells was significantly lower upon treatment with realgar as compared to arsenic trioxide, while orpiment showed an intermediate effect (Biocarta = 0, Kegg = 1, Hallmark = 6 for realgar; Biocarta = 31, Kegg = 44, Hallmark = 15 for arsenic trioxide: Biocarta = 0, Kegg = 17, Hallmark = 9 for orpiment) (Figure 5A; Table S2, Supporting Information).

The low off‐target effects of solubilized realgar on non‐APL cells at the pathway level were also mirrored when considering differentially expressed genes (DEGs, defined as FDR ≤ 0.05). Specifically, all treatments with arsenic‐containing compounds induced a higher number of DEGs in NB‐4 cells compared to Jurkat E6.1 cells (Figure 5B; Table S3, Supporting Information). However, while treatment with arsenic trioxide was associated with 235 upregulated and 240 downregulated DEGs in Jurkat E6.1 cells, realgar treatment induced only 4 DEGs in the same cell type (Table S3, Supporting Information), none of which displayed FDR < 0.01 (Figure 5B). Moreover, treatment of Jurkat E6.1 cells with orpiment led to an intermediate profile (Figure 5B; Table S3, Supporting Information), inducing 150 upregulated and 28 downregulated DEGs (Table S3, Supporting Information).

Taken together, these data indicate that solubilized realgar can preserve PML‐targeting and broad transcriptomic effects in APL cells while affecting a limited number of pathways and genes in non‐APL cells.

### 2.7 Anti HIV‐1 Latency Effects of Realgar

**Figure 6:**
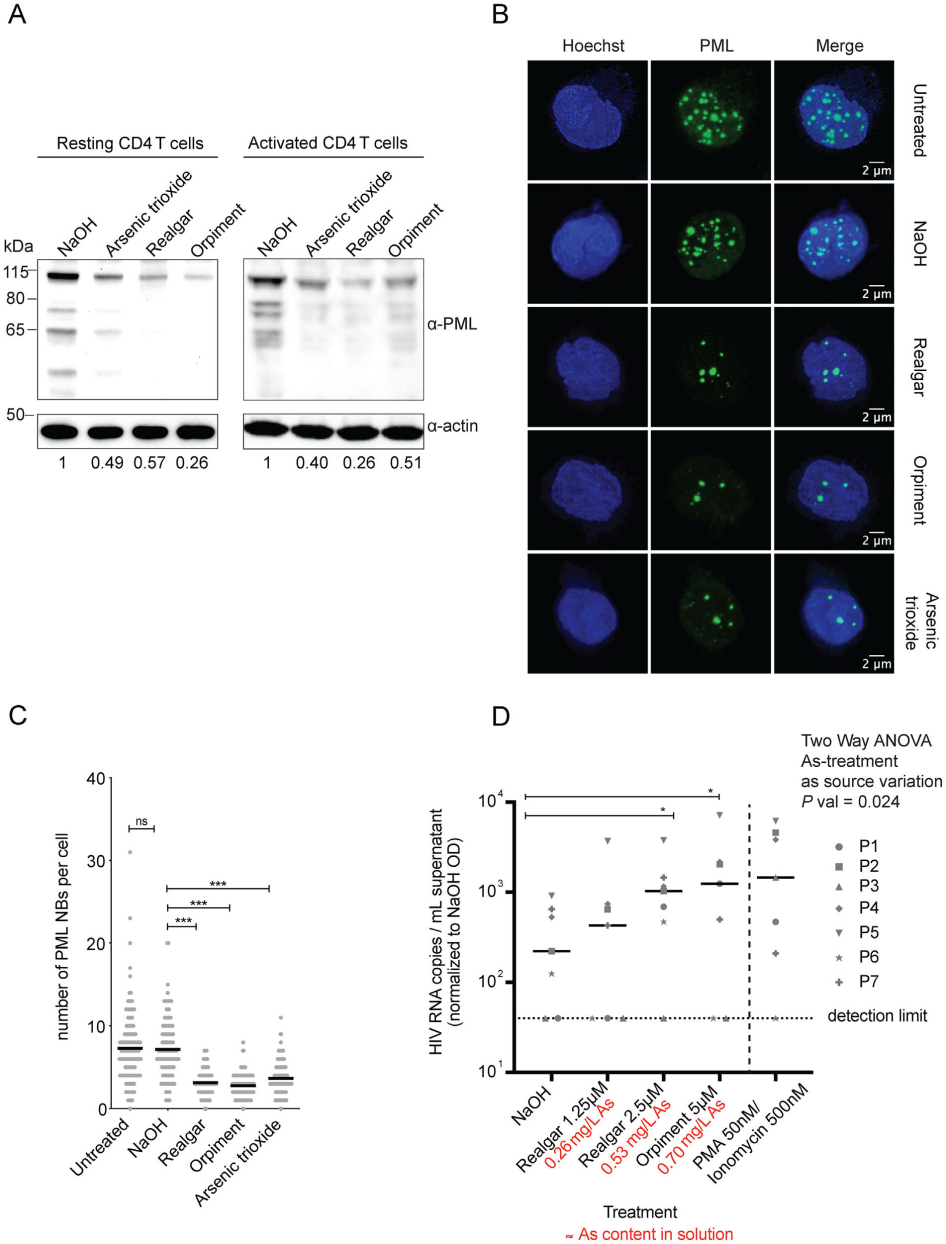
PML targeting and HIV latency reactivation potential of solubilized realgar in primary CD4 T cells. A–C) CD4 T cells were isolated from the total blood of healthy donors and treated for 24 h with 2 µm concentration of solubilized realgar (As content in solution = 0.42 mg L^−1^), orpiment, or arsenic trioxide (As content in solution = 0.28 mg L^−1^ for each). Cells were activated through treatment with αCD3‐CD28 beads, except in the left blot of panel A, where they were left resting. Treatment with NaOH was used as a vehicle control. A) Western blot analysis of PML protein levels. The numbers below each lane indicate the relative expression of PML, normalized to the housekeeping protein (actin) expression. B,C) Effect of solubilized realgar, arsenic trioxide or orpiment treatment on the degradation of PML NB as assessed by IF. Data in (B) were analyzed by the non‐parametric Kruskal‐Wallis test. D) Reactivation of HIV from latency in CD4 T cells of PLWH. Cells were isolated from the total blood of seven PLWH (Table S4, Supporting Information) and incubated with solubilized realgar or orpiment at the indicated concentrations, NaOH (solvent control), or PMA (50 nm) and ionomycin (500 nm) as a positive reactivation control. In all conditions, enfuvirtide (100 nm) was also added to prevent new cycles of infection. One week after treatment, HIV‐1 RNA levels were measured in the supernatant and normalized using relative MTT (Table S5, Supporting Information) OD values obtained at the same time point. Data were *Log10* transformed to restore normality and analyzed by two‐way ANOVA to detect the effect of arsenic‐containing treatments as source of variation. This was followed by the two‐stage linear step‐up procedure of Benjamini, Krieger and Yekutieli to estimate the impact of each arsenic containing drug dosage used. *p < 0.05; ***p < 0.001.

The PML‐depleting activity of arsenic trioxide has been previously associated with HIV‐1 latency reactivation in Jurkat‐derived cells and primary CD4 T cells.^[^ ^1^ ^]^ To estimate the potential of solubilized realgar as HIV latency reactivating agent, we first tested its ability to induce depletion of PML in primary CD4 T cells. Incubation with realgar led to PML depletion in both resting and activated primary CD4 T cells isolated from the total blood of healthy donors (**Figure** **6**A), without altering the expression of CD25 or CD69 activation markers in resting cells (Figure S5A,B, Supporting Information). Consistent with PML protein depletion, visualization of PML NBs by immunofluorescence in activated CD4 T cells revealed a significant reduction in their number in cells treated with solubilized realgar (Figure 6B,C). This is noteworthy as the number of PML NBs has previously been associated with maintenance of HIV‐1 latency.^[^ ^39^ ^]^

We then tested whether solubilized realgar and orpiment might share with arsenic trioxide a similar ability to induce HIV‐1 reactivation from latency. We first employed the well‐established latently infected T‐cell line J‐Lat 10.6, which is derived from Jurkat cells.^[^ ^19^ ^]^ J‐Lat cells harbor one copy of latent HIV‐1 DNA per cell, and viral reactivation can be detected through the expression of GFP. In the absence of any stimulation, only a small percentage (typically < 5%) of cells express HIV‐GFP (Figure S6, Supporting Information). After 48 h of treatment with realgar, GFP expression increased at the highest concentrations employed with limited effects on cell viability (Figure S6A,B, Supporting Information). Treatment with orpiment was associated with limited reactivation from latency, while arsenic trioxide was generally the most effective in reactivating HIV‐1 from latency (Figure S6B, Supporting Information), although it was associated with higher cytotoxicity (Figure S6A, Supporting Information), as also seen in our experiments treating uninfected Jurkat E6.1 cells (Figure 4B–D).

Finally, we investigated the effect of solubilized realgar in CD4 T cells isolated from the peripheral blood of seven donors living with HIV/AIDS (Table S4, Supporting Information) with viremias suppressed by ART, i.e., a model closer to the conditions occurring in vivo.^[^ ^40^ ^]^ Isolated CD4 T cells were treated with two different concentrations of realgar and one higher concentration of orpiment. As controls, we treated cells with NaOH (solvent control) or PMA/Ionomycin (positive control to induce latency reactivation). Enfuvirtide was also added to the medium in all conditions to prevent new cycles of infection and increase the likelihood that viral RNA copies detected in the supernatant are due to HIV‐1 reactivation from latency.^[^ ^41^ ^]^ Viral RNA levels were measured in supernatants a week after treatment, in line with previous literature.^[^ ^42^ ^]^ To account for drug effects on cell viability/proliferation, the levels of viral RNA were normalized to the relative MTT absorbance values (Table S5, Supporting Information) of each treated condition. Overall, the data showed that treatment with either solubilized orpiment or solubilized realgar were associated with an increase in the number of HIV‐1 copies in supernatant as compared to vehicle (NaOH) controls (Figure 6D), corroborating the potential anti‐HIV‐1 latency activity of arsenic‐containing compounds in general (as previously described for arsenic trioxide^[^ ^2^ ^]^) and of realgar in particular.

## 3 Discussion

The data presented herein describe a new approach to realgar solubilization, combining scalability and speed to yield a solution with biological activity in vitro and ex vivo. This solubilization process in part mimics the passage of realgar through the digestive system and might therefore yield some of the molecules to which realgar‐treated individuals are exposed.

Our protocol significantly deviates from any previously described transformation of realgar, occurring both in acidic media (whether electrochemically^[^ ^43^ ^]^ or bacterially mediated^[^ ^44^ ^]^) or through alkali.^[^ ^19^ ^]^ The difference between previous methods and the approach presented in this article is also supported by the chemical parameters we detected in solubilized realgar. For example, “classic” As‐S stretches have been described at 355–360 cm⁻¹ in homogeneous As‐S clusters. However, Zingaro et al. demonstrated, using different organic thioarsenides, that the frequency of the As‐S stretch may shift to the 400s cm⁻¹ when the arsenic atom is bound to a second moiety.^[^ ^24^ ^]^ The frequency may then vary depending on the substituent attached to the As atom.^[^ ^24^ ^]^ While this concept was established with organic moieties, there is no reason to exclude that the same phenomenon might also occur with inorganic ligands, whether covalently or coordinatively bonded, potentially altering the dipole properties of the As‐S bond. Therefore, the spectra presented here may provide an initial fingerprint of solubilized realgar, though the exact attribution of peaks in our Raman and MALDI‐TOF spectra remains uncertain and will require a dedicated study. Moreover, as early as in the 19th century, Berzelius observed a black precipitate after treating realgar with NaOH at 100°C, which he interpreted as metallic arsenic.^[^ ^19^ ^]^ We do confirm the presence of varying amount of a black precipitate that was significantly decreased, yet still present, after the third step of realgar solubilization. However, although our analyses support the presence of As_n_ clusters in the final precipitate, the Raman spectra cannot be explained solely with metallic arsenic and indicate additional atomic components.

Overall, the combined chemical analyses that we conducted show a high molecular diversity in the final realgar solution, comprising both As‐O and As‐S species and indicate that its composition is different from commercial preparations of solubilized arsenic trioxide and the realgar transforming solutions previously used to treat cells in vitro.^[^ ^17^, ^18^ ^]^

Our in vitro and ex vivo experiments indicate that submicromolar concentrations of solubilized realgar are sufficient to deplete PML in APL and non‐APL cells. This is in line with the extant literature, suggesting that PML might be degraded following the direct binding of As^3+^ ions,^[^ ^39^ ^]^ which are the active forms that bind to cellular thiols.^[^ ^45^ ^]^ These may be gradually released from the As‐containing chemical species in our solution. Our cytotoxicity and transcriptomic data indicate that solubilized realgar has significantly lower off‐target effects on the viability and transcriptional regulation of non‐APL cells compared to arsenic trioxide, even with a higher concentration of As equivalents. Solubilized realgar however maintains a selective activity in inducing the death of APL cells, thus suggesting that at least some of the arsenic species released from our process of realgar solubilization are less toxic than those deriving from arsenic trioxide. Arsenic trioxide was more potent than realgar in reactivating HIV‐1 from latency, but this effect was associated with higher cytotoxicity, suggesting that realgar might provide a balance between anti‐latency activity and maintenance of cell viability.

The present study also has some limitations. As mentioned above, the interpretation of the MALDI‐TOF peaks that we provide (Figure 2) is only tentative and more structural data will be necessary to confirm/define their chemical nature. Second, the different chemical species will need to be tested separately to determine an exact structure/activity relationship (SAR). This future task will, however, be very difficult as the different chemical species of arsenic can undergo rearrangements due to the different equilibria established after these species are isolated. The establishment of new equilibria or the re‐establishment of original ones^[^ ^46^ ^]^ thus renders the precise identification of the chemical species responsible for the biological effect very difficult. Moreover, our study only provides spectra of the chemical species contained in solubilized realgar with molecular weights above 138 Da due to the limitations of the techniques adopted. Finally, the concentrations of realgar required to impact HIV latency are in the micromolar range, which might be difficult to achieve in vivo. On the other hand, previous experiments in infected macaques have shown that arsenic trioxide can decrease the latent viral reservoir at well‐tolerated doses,^[^ ^2^ ^]^ suggesting that clinically utilized dosages may in any case target HIV latency, probably due to accumulation or time‐dependent phenomena. Given the recent clinical success of a realgar powder formulation in a phase III clinical trial enrolling people with APL,^[^ ^7^ ^]^ our solubilized realgar might be a candidate for quick translation to clinical testing if proven to be safe and effective in vivo. Further characterization of the realgar solutes might be important also in light of the recently described activity of realgar on arsenic trioxide‐resistant APL.^[^ ^47^ ^]^ While this activity remains anecdotal, its confirmation could open new therapeutic avenues for realgar. Identifying the specific realgar solutes responsible for overcoming arsenic trioxide resistance may also offer valuable molecular insights into drug‐resistant APL, paving the way for more targeted and personalized treatment approaches.

Improvement of safety and dosage increase might also be important to extend the therapeutic use of realgar beyond APL to chronic myeloid leukemia (CML), against which realgar is still an investigational agent in clinical trials.^[^ ^48^ ^]^ The potential activity on HIV latency merits attention given that dual treatment approaches for myeloid leukemias and HIV have previously resulted in remarkable clinical success.^[^ ^49^ ^]^

Overall, our results suggest a simple approach to solubilize realgar for studying its therapeutic properties and may open new avenues for the utilization of orally available arsenic‐containing compounds with an improved safety and efficacy profile.

## 4 Experimental Section

### Solubilization of Arsenic‐Containing Compounds

Realgar and orpiment were obtained in powder from Kremer Pigmente (Aichstetten, Germany). Arsenic trioxide was obtained from Sigma Aldrich (powder, ReagentPlus, ≥99.0%). To dissolve realgar, the powder was suspended at 100 g L^−1^ in an acidic aqueous solution containing HCl (pH 1–2) and incubated at room temperature for 24 h. The suspension was then diluted in 50‐mL Falcon tubes at a starting material/final volume ratio of 1:20 using a NaOH solution (pH 13–14) as a diluent and incubated at room temperature for 24 h. The suspension, including its dark precipitate, was thoroughly mixed and diluted 1:40 in NaOH (pH 12–13) in Falcon tubes, and incubated overnight at 65 °C. The same procedure was followed to solubilize orpiment or arsenic trioxide, except for the initial step in HCl. To assess the impact of highly alkaline conditions (pH 13–14) during the initial NaOH incubation, a control condition was included in which realgar was directly incubated in NaOH at pH 12–13.

### Fourier Transform Infrared (FT‐IR) Spectroscopy

FT‐IR spectra were obtained using a Bruker VERTEX 80v Spectrometer (Bruker, Germany) at room temperature. The spectra were recorded at a wavenumber resolution of 4 cm^−1^ using KBr pellets. Solutions deriving from each of the incubation steps described above were dried at 60°C in a thermostatically controlled heater in Eppendorf vials. An appropriate amount of sample was homogenized in agate mortar with KBr powder and then compressed into a pellet. The analyzed frequency range was 4000 to 400 cm^−1^. In order to get a high signal‐to‐noise ratio, 100 scans were run.

### Energy Dispersive X‐Ray Fluorescence Spectrometry

Energy dispersive X‐ray fluorescence *spectrometry* (ED‐XRF) measurements were carried out using an EDX‐720 instrument from Shimadzu (Japan), equipped with an X‐ray tube with an Rh target and a Si(Li) detector, as previously described.^[^ ^50^, ^51^, ^52^ ^]^ The operating conditions were: voltage = 15–50 kV; current = auto; collimator = 10 mm; X‐ray atmosphere = air; measurement time = 60 s. Typically, 5 mg of solid samples or 200 µL of liquid samples were placed inside the plastic support upon the Mylar film, and the analysis was performed by monitoring the Kα line for sulfur (2.1 – 2.5 KeV) and the Kα line for arsenic (10.3 – 10.7 KeV).

### Raman Spectroscopy

Raman spectroscopy analysis was performed using a Witec Alpha 300R confocal microscope (Germany) with an excitation wavelength of 633 nm and equipped with a piezoelectric table for sample deposition. The objective lenses (Nikon) of 10x (NA 0.25) and 20x (NA 0.4) were used to focus the samples. A pinhole of 100 µm was used with a CCD detector.

All samples were analyzed in liquid form, with the solution deposited in a polypropylene sample holder. The laser intensity was set near the maximum intensity without causing sample burn. The spectrum was obtained with an integration time of 15 s and 360 accumulations, followed by further treatment for cosmic ray removal and baseline corrections through the WITec suite (Oxford Instruments).

### Mass Spectrometry

Mass spectrometry (MS) was initially performed in a Shimadzu LCMS‐2020, equipped with an ESI (ElectroSpray Ionization) source and a triple‐quadrupole analyzer, in direct injection mode and using a mobile phase of ammonium formate (50 mmol L^−1^). Mass spectra were analyzed in both positive and negative modes. All samples were analyzed in aqueous solution form, with an injection volume of 10 µL and a total analysis time of 3 min. Further mass spectrometry analyses were performed with a Bruker MALDI Ultraflextreme Bruker Daltonics, using a MALDI (Matrix‐Assisted Laser Desorption/Ionization) source and a Time‐of‐Flight (TOF) analyzer. Data acquisition was carried out in MS and MS/MS modes using laser power ≈ 20% and DHB (2,5‐dihydroxybenzoic acid) as the ionization matrix.

### Inductively Coupled Plasma Mass Spectrometry (ICP‐MS)

Arsenic concentrations in arsenic trioxide, realgar, and orpiment were analyzed using inductively coupled plasma mass spectrometry (ICP‐MS), optimized for the quantitative determination of heavy metals in aqueous samples. The measurements were conducted at the Center for Infectiology, Chemistry Laboratory, Section of Hospital and Environmental Hygiene, University Hospital Heidelberg. Samples were prepared by diluting 1:2000 in a 1% HNO_3_ solution and the analysis was performed on an ICP‐MS device Analytik Jena PlasmaQuant MS. A calibration curve was established using commercially available certified reference materials, including arsenic standard solutions. Specifically, the ICP Multi‐Element Standard Solution (28 elements, 1 mg L^−1^ each of: Al, Ag, As, B, Ba, Be, Bi, Ca, Cd, Co, Cr, Cu, Fe, K, Li, Mg, Mn, Mo, Na, Ni, Pb, Sb, Se, Sr, Ti, Tl, V, Zn, all in 2% HNO3) obtained from ROTH, Germany (Art. No. 2649.1), was utilized for the analysis.

### Cell Lines

Jurkat E6.1 cells were obtained through the European Collection of Authenticated Cell Cultures (ECACC, Catalog no. 88042803). The HIV‐infected Jurkat‐derived cell line J‐Lat 10.6^[^ ^53^ ^]^ was obtained through the NIH AIDS Reagent Program. The acute promyelocytic leukemia cell line NB‐4 was obtained from Cell Lines Service (Cat: 300299; used for Western blot experiments) and as a kind gift from Dr. Borko Amulic (University of Bristol; used for cytotoxicity and RNA‐Seq experiments). These cell lines were cultured in RPMI 1640 medium supplemented with 10% fetal bovine serum (FBS) and 1% penicillin/streptomycin in an incubator at 37 °C in a 5% CO_2_ atmosphere. The human melanoma cell line A‐375 was obtained from the American Type Culture Collection (ATCC CRL‐1619) and cultured in Dulbecco's Modified Eagle Medium (DMEM) supplemented with 10% FBS, penicillin (100 mg mL^−1^), and streptomycin (100 mg mL^−1^) in an incubator at 37 °C in a 5% CO_2_ atmosphere. The human colorectal adenocarcinoma cell line SW480 (ATCC – CCL‐228) was cultured in Leibovitz's L‐15 Medium supplemented with 100 U mL^−1^ penicillin 100 µg mL^−1^, streptomycin, and 10% FBS in an incubator at 37 °C without CO_2_.

### Primary Cells

Human umbilical vein endothelial cells (HUVEC; ATCC CRL‐1730), were cultivated in RPMI 1640 medium supplemented with 2.38 g L^−1^ of Hepes and 10% FBS in an incubator at 37 °C in a 5% CO_2_ atmosphere.

Immortalized cancer associated human fibroblast cells (HF002J cells), were kindly provided by Prof. Monica Beatriz Mathor of the Instituto de Pesquisas Energéticas e Nucleares – IPEN USP, following project approval by the local ethics committee (CAAE 10867212.3.0000.5421). HF002J cells were maintained in RPMI 1640 medium supplemented with 10% FBS, penicillin/streptomycin, at 37 °C in a 5% CO_2_ atmosphere.^[^ ^54^ ^]^

Whole blood from healthy donors was obtained from the Heidelberg University Hospital Blood Bank following approval by the local ethics committee (Project ID: S‐604/2020). CD4 T cells were isolated from the whole blood of healthy donors using the RosetteSep Human CD4 T Cell Enrichment Cocktail (STEMCELL Technologies Inc., Canada) following the manufacturer's instructions. To activate cells after isolation, Dynabeads® Human T‐Activator CD3/CD28 (Thermo Fisher Scientific) was added for 48 h using a bead/cell ratio of 1:5. Cells were kept in RPMI 1640 medium supplemented with 20% FBS, 1% penicillin/streptomycin, and 10 ng mL^−1^ IL‐2 at 37 °C in a 5% CO_2_ atmosphere.

The use of samples from people living with HIV (PLWH) was approved by the Human Subjects Review Committee of the Federal University of São Paulo (Project ID: 83012617.8.0000.5505). All subjects gave written informed consent. CD4 T cells were isolated from the total blood of adults under suppressive antiretroviral therapy (ART) for at least 12 months, and with low or undetectable viral loads (HIV‐1 RNA levels < 50 copies per mL of plasma). The characteristics of the PLWH enrolled in the study are summarized in Table S4 (Supporting Information). Resting CD4 T cells were isolated by negative selection from whole blood of PLWH using the EasySep RosetteSep Human CD4 T cell isolation kit (STEMCELL Technologies Inc., Canada), according to the manufacturer's instructions.

### Drug Treatments

Cells were treated with different concentrations of solutions of realgar, orpiment, arsenic trioxide, or vehicle control (NaOH pH 12–13). Phorbol 12‐myristate 13‐acetate (PMA; 50 nM) (Cambridge Bioscience), alone or in combination with ionomycin (500 nM) (Sigma Aldrich), was used as a positive control in HIV reactivation experiments.

The treatment duration was 24 h for the collection of primary CD4 T cells and NB‐4 cells for subsequent protein extraction, fluorescence‐activated cell sorting (FACS), and immunofluorescence (IF) analysis; 48 h for RNA extraction for RNA‐Seq in Jurkat E6.1 and NB‐4 cells and for measuring the reactivation of HIV expression in J‐Lat 10.6 cells; and 7 days for measuring the reactivation of HIV expression in primary CD4 T‐cells of PLWH. During treatment, the medium of cells from PLWH was further supplemented with 100 nM enfuvirtide (T‐20) to prevent new cycles of infection. Time course analyses of cytotoxicity were performed at 24, 48, and 72 h.

### Cell Viability

Cell viability was assessed by using a 5 mg mL^−1^ solution of MTT in water (Sigma Aldrich) as previously described.^[^ ^55^ ^]^ Briefly, cells (0.1 × 10^6^ in 100 µL) were plated into a flat‐bottom 96‐well plate, and 15 µL of MTT was added to each well at the end of the drug incubation period. After 2–4 h of incubation in the dark at 37 °C in a 5% CO_2_ atmosphere, the reaction was stopped by adding 100 µL of 10% SDS in water. Absorbance was measured using a GloMAX® Explorer (Promega) or a SpectraMax Plus (Molecular Devices) microplate reader.

### Adhesion Assay

Cells were trypsin‐detached from flasks after growing for three days, washed three times in PBS and seeded at a concentration of 5000 cells mL^−1^ in 96‐well plates in the presence or absence of the test drugs. After 24 h, microphotographs were taken from at least five randomly selected fields using an inverted light microscope (Axio Observer A1, Carl Zeiss, Jena, Germany).

### FACS Analysis

For FACS analysis, 0.5–1 × 10^6^ cells were stained and analyzed using either a FACS Celesta or FACSVerse (BD Bioscience, USA), or a Novocyte 3000 machine (Agilent Technologies). Cells were pelleted at 1000 rpm at room temperature and, after washing in PBS, stained for 10 min with a LIVE/DEAD dye (either Zombie Far Red or Green Fixable Viability Kit, BioLegend, USA, 1:1000 in PBS). Cells were then washed in PBS and fixed with 4% PFA. For J‐Lat 10.6 cells, GFP levels were acquired on a Novocyte 3000 machine. The viability of primary (resting or activated) CD4 T cells was analyzed on a FACS Celesta machine. To analyze activation markers, CD4 T cells were stained with CD69 or CD25 (APC conjugated, BD Bioscience, USA) antibodies in the dark for 30 min on ice. Cells were then washed twice with FACS buffer (2% FBS in PBS) before being analyzed on a FACSVerse machine. Data was analyzed using FlowJo software (BD Biosciences).

### SDS‐PAGE and Western Blot

Cells were harvested and homogenized on ice for 10 min in 1x Radioimmunoprecipitation assay (RIPA) buffer (50 mm Tris HCl pH 8, 150 mm NaCl, 0.5% Sodium Deoxycholate, 0.5% IGEPAL CA‐630, 0.1% SDS) supplemented with protease (cOmplete Tablets Mini EDTA‐free EASYpack, Sigma Aldrich, USA) and phosphatase (PhosSTOP EASYpack, Roche) inhibitors. For further lysis, cells were sonicated (Sonorex Super RK102H, Sigma Aldrich, USA) for 5 min (30 s on/30 s off on ice). Protein concentration was assessed using the Micro BCA Protein Assay Kit (Thermo Fisher Scientific), measuring absorbance at 562 nm on an Infinite 200 PRO multimode plate reader (Tecan, Switzerland). Samples were boiled at 95 °C for 5 min and then loaded onto a 4–12% NuPAGE gel (Thermo Fisher Scientific) with MOPS SDS Running Buffer. Proteins were transferred onto a nitrocellulose membrane (Carl Roth GmbH) for visualization by wet‐transfer Western blot analysis, applying 20 V for 2 h at room temperature. Membranes were then blocked in PBS‐Tween containing 5% milk for 1 h at room temperature, and subsequently incubated overnight at 4 °C with primary antibodies directed against PML (PML A301‐167A, rabbit; Bethyl Laboratories, Montgomery, USA) and actin (anti‐beta actin, a2228, mouse; Sigma Aldrich, St. Louis, USA) diluted, respectively, 1:500 and 1:5000 in 0.01% PBS‐T containing 5% milk. After washing, membranes were incubated with secondary antibodies α‐mouse and α‐rabbit IgG HRP (1:5000, Jackson ImmunoResearch Laboratories, West Grove, PA, USA). Western blots were developed using SuperSignal™ West Pico PLUS Chemiluminescent Substrate (Thermo Fisher Scientific). For re‐probing the membrane, a stripping buffer containing 8% upper gel buffer (0.5 M TRIS‐HCl pH 6.8, 0.4% SDS), 2% SDS, 110 mM β‐mercaptoethanol was used for 4 times × 5 min at 65 °C with shaking.

### Immunofluorescence (IF)

Cells were plated on polyethylenimine (PEI)‐coated coverslips placed into a 24‐well plate at 37 °C for 1 h, subsequently rinsed with PBS, and fixed in 4% PFA in PBS for 10 min. Coverslips were then washed three times for 5 min with PBS. To permeabilize the cells, they were incubated with 0.5% Triton X‐100/PBS (PBS‐T) for 10 min. After washing the coverslips again three times for 5 min with 0.1% PBS‐T, they were blocked with 4% BSA/PBS for 45 min at room temperature to prevent nonspecific binding and subsequently incubated with primary antibodies against PML (described in the Western blot section above). The coverslips were then washed three times with 0.1% PBS‐T and incubated with the secondary antibodies Alexa‐488 α‐rabbit (Thermo Fisher Scientific, Karlsruhe, Germany) at a 1:1000 dilution for 1 h at room temperature. Following three more washing steps, nuclear counterstaining was performed using Hoechst 33342 in PBS (1:10,000) for 5 min. Coverslips were washed again twice in PBS and mounted with Mowiol. Data were acquired using a Leica TCS SP8 confocal microscope with a 63x oil immersion objective (Leica Microsystems GmbH, Germany). A z‐step of 500 nm was used to attain 3D stacks with a zoom set at 3x. To count the number of PML NBs, the TCS SP8 setting was used. To acquire higher resolution images for visualization, the Lightning setting was used.

### PML Nuclear Bodies Image Analysis

PML NBs were analyzed using the Image J macro reported previously.^[^ ^39^ ^]^ Briefly, cells were identified and counted using the Hoechst‐stained images that were enhanced for contrast (saturated = 0.35) and smoothed using a median filter (radius = 3). Otsu's thresholding method was applied to segment the nuclei (threshold: 75–255). The resulting binary image was projected to maximum intensity across the Z‐stack. A watershed algorithm was then applied to separate touching nuclei. Finally, particles were analyzed with size constraints of 3‐Infinity pixels and circularity of 0.40‐1.00, excluding edges and including holes. The results were summarized and added to the ROI manager. For subsequent counting of PML NBs, cells were further segmented. The image intensity was adjusted (min = 0, max = 150), followed by Gaussian blur (sigma = 2) applied to the stack. RenyiEntropy thresholding was used with a threshold range of 80–255. The resulting binary mask represented the segmented cells, with the ROI manager showing all identified cell regions. A duplicate of the segmented image was created for PML NB counting. The 3D Objects Counter plugin was utilized with a threshold of 1, analyzing the 16th slice of the stack. Objects were counted with size constraints between 1 and 356,796 voxels. The plugin generated object statistics and a summary of the count results.

### RNA‐Seq

NB‐4 and Jurkat E6.1 cells treated for 48 h with 0.5 µm realgar, 0.5 µm arsenic trioxide, 0.5 µm orpiment or solvent (NaOH) were used for total RNA extraction using the SV Total RNA Isolation System (Promega #Z3100) according to manufacturer's instructions.

RNA‐Sequencing was performed by Novogene. Briefly, mRNA was purified from total RNA using poly‐T oligo‐attached magnetic beads. After fragmentation, the first strand cDNA was synthesized using random hexamer primers followed by the second strand cDNA synthesis. The library was ready after end repair, A‐tailing, adapter ligation, size selection, amplification, and purification, and was quality checked by Qubit and real‐time PCR for quantification and by bioanalyzer for size distribution detection. Paired‐end sequencing was performed through Illumina Sequencing PE150. Raw reads were filtered to remove adapters, reads containing undetermined (N) bases > 10% and reads containing low quality (Qscore≤ 5) bases over 50% of the total base.

Raw reads were aligned using STAR (version 2.7.9a)^[^ ^56^ ^]^ to build version hg38 of the human genome. Counts for UCSC annotated genes were calculated from the aligned reads using the featureCounts function of the Rsubread R^[^ ^57^ ^]^ package and R (version 4.1.1). Normalization and differential analysis were carried out using the edgeR R package.^[^ ^58^ ^]^ Raw counts were normalized to obtain Counts Per Million mapped reads (CPM). Only genes with a CPM greater than 1 in at least 3 samples were retained for differential analysis. Pathway over‐representation analysis was performed by Gene Set Enrichment Analysis (GSEA, version 4.2.2)^[^ ^38^ ^]^ in preranked mode and using the gene sets of the Hallmark, Kegg and Biocarta collections from the Broad Institute Molecular Signatures Database (http://software.broadinstitute.org/gsea/msigdb). Gene sets were considered significantly enriched at FDR < 5% when using Log2 fold change as a metric and 1,000 permutations of gene sets.

The RNA‐Seq data from this study have been deposited at Gene Expression Omnibus database (GEO, https://www.ncbi.nlm.nih.gov/geo/) with accession number GSE270992.

### Quantification of Viral Load in Supernatants

Viral reactivation in cells isolated from PLWH was assessed using 1 mL of cell culture supernatant, as previously described.^[^ ^59^ ^]^ Briefly, viral RNA in supernatants was quantified using an Abbott RealTime HIV‐1 Viral Load Assay (Abbott Laboratories, Lake Forest, IL, USA) running on an automated RNA extraction and detection platform m2000 (Abbott Laboratories) according to the manufacturer's instructions. The detection limit of the assay is 40 copies mL^−1^.

### Statistical Analyses and Data Visualization

Statistical analyses were performed using GraphPad Prism, v. 10.2.3 (GraphPad Software, Inc., Boston, MA). Differences between variables were assessed using paired *t*‐tests (threshold: *P*  = 0.05), or, in case of multiple comparisons, using the Kruskal‐Wallis non‐parametric test, or one‐ or two‐way ANOVA. The transformations adopted to restore normality are specified in the figure captions. Drug treatments were performed at least in duplicate and repeated on three separate occasions. CC50 values were calculated by non‐linear regression using the *Log inhibitor vs. normalized response* function embedded in the GraphPad software. The possibility that one curve might adequately describe all data sets (null hypothesis) was analyzed by the extra sum‐of‐squares *F* test, using the LogIC50 as a parameter of comparison, and a threshold *P* value lower than 0.05 for exclusion of the null hypothesis.

Graphs were generated in GraphPad Prism except for heatmaps, bar plots, and volcano plots which were created using RStudio (v2024.04.2, Posit Software, PBC, Boston, MA, USA).

## Conflict of Interest

B.L., M.E.H., M.L., I.L.S., and A.S. are inventors of a patent application covering the therapeutic use of solubilized realgar.

## Author Contributions

I.L.S. and A.S. contributed equally to this work. B.L., M.E.H., M.L., I.L.S., and A.S. conceived the study. B.L., D.S.F., M.L., I.L.S., and A.S. designed experiments. B.L., D.S.F., H.P.N., L.G., A.P.S.J., T.D., C.G., J.K., A.M., M.R., J.F.S., I.L.S., and A.S. planned and performed experiments. M.T. M.H.E., and H.M. provided cells and/or reagents. B.L., D.F., L.G., A.M., M.T., H.M., M.L., I.L.S., and A.S. interpreted and discussed results. M.T. and A.S. conducted the molecular modeling. B.L., M.F., I.L.S., and A.S. analyzed the data. A.S. directed the study. M.L. and I.L.S. co‐directed the study. I.L.S. and A.S. wrote the manuscript. All authors have read and approved the manuscript.

## Supporting information



Supporting Information

Supplemental Table 1

Supplemental Table 2

## Data Availability

RNA‐Seq data are deposited under the GEO accession GSE270992. All other relevant data supporting the key findings of this study are available within the article and its supplementary information files or from the corresponding author on reasonable request.
