# Supplementary material for: Fast pH‐Driven Solubilization Method of Realgar (As4S4) to Reduce the Toxicity of Arsenic [As(III)] for Medicinal Purposes
Source: Adv Sci (Weinh). 2025 Apr 24;12(29):2502740. doi: 10.1002/advs.202502740 (PMC12362784; doi:10.1002/advs.202502740)
Supplement: Supplementary file 1 — Supporting Information [file ADVS-12-2502740-s003.docx]

**Supporting Information**

**Fast pH-Driven Solubilization Method of Realgar (As_4_S_4_) to Reduce the Toxicity of Arsenic [As(III)] for Medicinal Purposes**

Bojana Lucic^1,2^, Douglas Santana Franciscato^3^, Helton Pereira Nogueira^3^, Lara Gallucci^4^, Alceu Totti Silveira Junior^3^, Asmaa Mohamed Ismail^5^, Millie Robinson^4^, Teresa Dallinger^1^, Claudia Gutfleisch^6^, Jochen Kurz^6^, Maytê Toledo^7^, Jessica Dias da Silva Ferraz^8^, Mohammad Tarek^9^, Danilo Dias^8^, Ricardo Sobhie Diaz^8^, Mahmoud ElHefnawi^10^, Mattia Forcato^11^, Hugo P Monteiro^7^, Marina Lusic^1,2^, Iart Luca Shytaj^4,8^*^§^, Andrea Savarino^12^*^§^.

1 Department of Infectious Diseases, Integrative Virology, Heidelberg University, Heidelberg, 69120, Germany.

2 German Center for Infection Research, Heidelberg, 69120, Germany.

3 Institute of Chemistry, University of São Paulo, São Paulo, 05508-220, Brazil.

4 School of Cellular and Molecular Medicine, University of Bristol, Bristol, BS8 1TD, UK.

5 Spectroscopy Department, National Research Centre, 33 El Bohouth Street, Dokki, 12622 Giza, Egypt.

6 Center for Infectious Diseases, Medical Microbiology und Hygiene, University Hospital Heidelberg, Heidelberg, 69120, Germany

7 Department of Biochemistry, Center for Cellular and Molecular Therapy, Federal University of São Paulo, São Paulo, 04021-001, Brazil.

8 Infectious Diseases Department, Federal University of São Paulo, São Paulo, 04021-001, Brazil.

9 Clinical Hematology Department, Armed Forces College of Medicine (AFCM), Heliopolis, Cairo Governatorate, 11774, Egypt.

10 Informatics and Systems Department, National Research Centre, 33 El Bohouth Street, Dokki, 12622 Giza, Egypt.

11 Department of Molecular Medicine, University of Padova, Padova, 35122, Italy.

12 Department of Infectious Diseases, Italian Institute of Health, Rome, 00161, Italy.

*Equal contribution

^§^ Correspondence:

[andrea.savarino@iss.it](mailto:andrea.savarino@iss.it)

[luca.shytaj@bristol.ac.uk](mailto:luca.shytaj@bristol.ac.uk)

**Supplementary figures**

**Supplementary Figure 1.** **FT-IR characterization of the products of realgar solubilization steps**. The curves show the FT-IR spectra of realgar in powder form (black) and of the dried material derived from steps 1 (HCl, red), steps 2-3 (NaOH pH 13-14, blue), and step 4 (NaOH pH 12-13, green) of the solubilization protocol described in Figure 1. The vibrational bands at 3435 cm^−1^ and 1641 cm^−1^ are attributed to the absorption of O-H stretching and bending vibrations corresponding to the moisture in air. The FT-IR spectrum of orpiment in powder form (orange) is shown as a matter of comparison.

**Supplementary Figure 2**. **Semi-quantitative ED-XRF analysis of different steps of realgar and orpiment solubilization.** Supernatants (light blue for arsenic and white for sulfur) and particulate matter (purple for arsenic and violet for sulfur) were analyzed separately. Note that the sulfur content is underestimated because its low atomic number approximates the detection threshold of the technique. Supernatants and particulate matter of orpiment are shown as a matter of comparison.

**Supplementary Figure 3. Comparative Raman spectroscopy analysis of solubilized realgar.** The solution and small amount of black precipitate obtained after the final step of realgar solubilization (step 4 in Figure 1) were analyzed by Raman spectroscopy. The figure depicts the comparison between the Raman spectra of solubilized realgar (A) and black particulate (B) with reference spectra for sodium arsenate (C) and sodium arsenite (D). *x* axis: Raman shift (cm⁻¹). *y* axis: normalized intensity (a.u.). All Raman spectra are normalized using the most intense peak as reference (=1).

Reference spectra for arsenates and arsenites were retrieved from: SpectraBase Compound ID: DcgQIspdvbW, and SpectraBase Compound ID: 606PKSOrnxm, respectively.


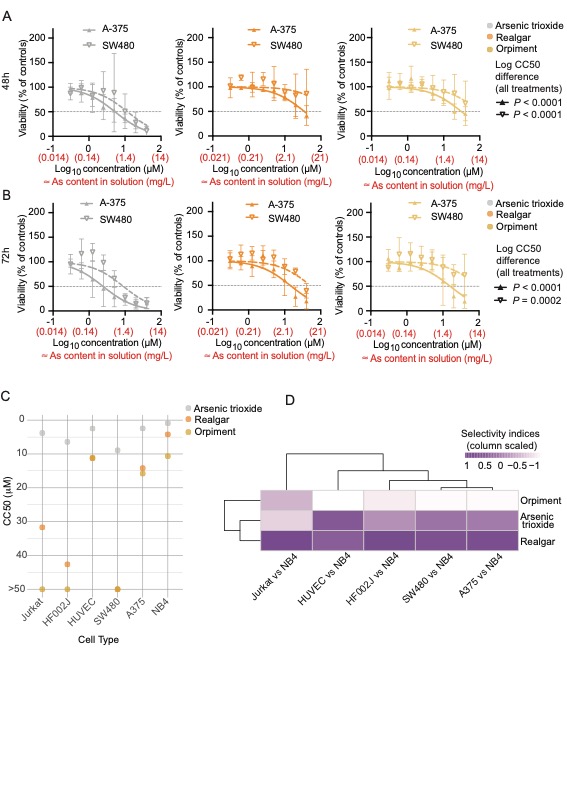


**Supplementary Figure 4. Cytotoxic effects of arsenic-containing compounds on cell lines and primary cells.** (A-B) Viability of A-375 melanoma cells and SW480 colon carcinoma cells after 48 hour- (A) and 72 hour- (B) treatment with arsenic-containing compounds. Cell viability was assessed by MTT assay after treatment with serial dilutions of arsenic trioxide, realgar, or orpiment solutions. Absorbance values were normalized to NaOH (solvent) control. Half-maximal cytotoxic concentration (CC50) values were calculated by non-linear regression. Extra sum-of-squares *F* test was performed to assess differences in LogCC50 values between the inhibition curves. Data are depicted as mean ± SD. N = 3. (C,D) The CC50 values at 72 hours post-treatment (C), derived from panel B and Figures 3E, 4D, were used to generate a heatmap (D) of selectivity indices for the cytotoxicity of arsenic-containing compounds towards APL cells. Selectivity indices were calculated as the CC50 towards each indicated cell type/CC50 in NB-4 cells. CC50 values were capped at 50µM to reflect the drug concentration range used in their determination. Note that the y axis in C is inverted in line with the inverse relationship between CC50 and cytotoxicity. Z-score normalization (column scaling) was applied to the heatmap in (D). The dotplot in (C) and the heatmap in (D) were generated using the ggplot2 and pheatmap packages in Rstudio, respectively.

**Supplementary Figure 5. Effect of arsenic-containing compounds on T cell activation markers.** CD4 T cells were isolated from the total blood of healthy donors and treated for 24 hours with 2 μM concentration of solubilized arsenic trioxide, realgar or orpiment. Treatment with NaOH was used as a vehicle control, while treatment with αCD3-CD28 beads was used as a positive activation control. The panels depict flow cytometry analysis of the expression of CD25/IL2R (A) and CD69 (B).

**Supplementary Figure 6. Effect of arsenic-containing compounds on cell viability and HIV latency reactivation in Jurkat-derived cells.** J-Lat 10.6 cells were treated with serial dilutions of solubilized realgar, arsenic trioxide, and orpiment. Treatment with NaOH was used as a vehicle control, while treatment with PMA (50 nM) was used as a positive control inducing HIV-1 reactivation from latency. After 24 hours, cells were stained with a LIVE/DEAD Zombie dye and analyzed by flow cytometry for cell viability (A) and GFP-HIV expression (B). Lines represent median values. Note the break in the Y axis in (B) to allow better comparison between arsenic-containing compounds.

**Supplementary tables**

**Supplementary Table 1. ICP-MS determination of As content in arsenic containing solutions.** Realgar was solubilized as outlined in Figure 1, while arsenic trioxide and orpiment were dissolved in the same NaOH buffer used in the final step of the realgar solubilization protocol. For each solution, 1 mL was analyzed to determine the arsenic atom content using ICP-MS.

| **Compound** | **Molarity (µM)** | **MW** | **Estimated As concentration (mg/mL)** |
| --- | --- | --- | --- |
| Arsenic Trioxide (As_2_O_3_) | 630 µM | 197.8 | 85.2 mg/L |
| Realgar (As_4_S_4_) | 292 µM | 427.9 | 61.4 mg/L |
| Orpiment (As_2_S_3_) | 508 µM | 246 | 70.7 mg/L |

**Supplementary Table 2. List of transcriptomic pathways modulated by realgar or arsenic trioxide in Jurkat E6.1 and NB-4 cells.** Jurkat E6.1 (non-APL) and NB-4 (APL) cells were treated for 24 hours with 0.5 µM of either realgar, arsenic trioxide, or solvent (NaOH) and subjected to RNA-Seq. The table shows the list of pathways retrieved from the annotated gene set repositories Hallmark, Biocarta, and KEGG and tested by GSEA, comparing each treated condition with the corresponding solvent control. Significantly modulated pathways (FDR ≤ 0.25) are highlighted.

**Supplementary Table 3. List of differentially expressed genes following treatment with realgar or arsenic trioxide in Jurkat E6.1 and NB-4 cells.** Jurkat E6.1 (non-APL) and NB-4 (APL) cells were treated for 24 hours with 0.5 µM of either realgar, arsenic trioxide, or solvent (NaOH) and subjected to RNA-Seq. The table shows the list of differentially expressed genes (DEGs, *i.e.*, genes with FDR ≤ 0.05) obtained by comparing each treated condition with the corresponding solvent control using the edgeR package.

**Supplementary Table 4. Demographic characteristics of PLWH donating blood for *ex vivo* latency reversal experiments.** VL = HIV-1 viral load.

| **ID** | **Age** | **Sex** | **ART** | **VL (copies/mL of plasma)** | **Year of diagnosis** | **CD4 counts (cells/mm^3^)** |
| --- | --- | --- | --- | --- | --- | --- |
| P1 | 48 | F | dolutegravir/lamivudine/darunavir-ritonavir | Undetectable | 1998 | 397 |
| P2 | 35 | M | dolutegravir/tenofovir/lamivudine | Undetectable | 2006 | 753 |
| P3 | 50 | M | biovir (i.e. lamivudine + zidovudine), atazanvir, ritonavir, tenofovir | Undetectable | 1994 | 1339 |
| P4 | 54 | M | dolutegravir/tenofovir/lamivudine | Undetectable | 2009 | 700 |
| P5 | 36 | M | dolutegravir/lamivudine/abacavir | 44 copies/mL | 2012 | 1205 |
| P6 | 29 | M | dolutegravir/tenofovir/lamivudine | Undetectable | 2013 | 943 |
| P7 | 45 | M | dolutegravir/tenofovir/lamivudine | Undetectable | 2009 | 668 |

**Supplementary Table 5. MTT absorbance coefficients used to normalize HIV expression levels in the supernatants of CD4 T cells isolated from the blood of PLWH.** CD4 T cells were isolated from the total blood of seven PLWH (Supplementary Table 1) and incubated with solubilized realgar, solubilized orpiment, NaOH (solvent control), or PMA (50 nM) and ionomycin (500 nM). In all conditions, Enfuvirtide (100 nM) was also added to prevent new cycles of infection. One week after treatment, cells were collected and assayed by MTT. The table depicts the ratios between absorbances at 570 nm of cells treated with realgar or PMA/ionomycin vs. NaOH. The ratios depicted in the table were used to normalize HIV-1 RNA levels detected in the supernatant (Figure 6D).

| **ID** | **OD Ratios by treatment (normalized to NaOH OD)** | | | |
| --- | --- | --- | --- | --- |
|  | **realgar 2.5μM (≈ 0.53 mg/mL As in solution)** | **realgar 1.25μM (≈ 0.26 mg/mL As in solution)** | **orpiment 5μM (≈ 0.70 mg/mL As in solution)** | **PMA 50nM/Ionomycin 500nM** |
| P1 | 0.56 | 0.74 | 0.43 | 3.57 |
| P2 | 0.40 | 0.48 | 0.32 | 1.35 |
| P3 | 0.67 | 0.72 | 0.30 | 1.35 |
| P4 | 0.69 | 0.83 | 0.55 | 1.85 |
| P5 | 0.41 | 0.66 | 0.24 | 1.57 |
| P6 | 0.52 | 0.70 | 0.31 | 1.42 |
| P7 | 0.46 | 0.66 | 0.28 | 1.64 |
